# Preventive effects of the sodium glucose cotransporter 2 inhibitor tofogliflozin on diethylnitrosamine-induced liver tumorigenesis in obese and diabetic mice

**DOI:** 10.18632/oncotarget.16874

**Published:** 2017-04-06

**Authors:** Koki Obara, Yohei Shirakami, Akinori Maruta, Takayasu Ideta, Tsuneyuki Miyazaki, Takahiro Kochi, Hiroyasu Sakai, Takuji Tanaka, Mitsuru Seishima, Masahito Shimizu

**Affiliations:** ^1^ Department of Gastroenterology, Gifu University Graduate School of Medicine, Gifu, Japan; ^2^ Department of Informative Clinical Medicine, Gifu University Graduate School of Medicine, Gifu, Japan; ^3^ Department of Pathological Diagnosis, Gifu Municipal Hospital, Gifu, Japan

**Keywords:** sodium glucose cotransporter 2 (SGLT2), hepatocellular carcinoma (HCC), non-alcoholic fatty liver disease (NAFLD), obesity, diabetes mellitus

## Abstract

Sodium glucose cotransporter 2 inhibitors are expected to ameliorate the abnormalities associated with metabolic syndrome including non-alcoholic fatty liver disease. In this study, we investigated the effects of the sodium glucose cotransporter 2 inhibitor tofogliflozin on the development of non-alcoholic fatty liver disease-related liver tumorigenesis in C57BL/KsJ-+Lepr*^db^*/+Lepr*^db^* obese and diabetic mice. The direct effects of tofogliflozin on human liver cancer cell proliferation were also evaluated. Mice were administered diethylnitrosamine-containing water for 2 weeks and were treated with tofogliflozin throughout the experiment. In mice treated with tofogliflozin, the development of hepatic preneoplastic lesions was markedly suppressed, and hepatic steatosis and inflammation significantly reduced, as evaluated using the non-alcoholic fatty liver disease activity score, in comparison with the control mice. Serum levels of glucose and free fatty acid and mRNA expression levels of pro-inflammatory markers in the liver were reduced by tofogliflozin treatment. Conversely, the proliferation of sodium glucose cotransporter 2 protein-expressing liver cancer cells was not inhibited by this agent. These findings suggest that tofogliflozin suppressed the early phase of obesity- and non-alcoholic fatty liver disease-related hepatocarcinogenesis by attenuating chronic inflammation and hepatic steatosis. Therefore, sodium glucose cotransporter 2 inhibitors may have a chemopreventive effect on obesity-related hepatocellular carcinoma.

## INTRODUCTION

Hepatocellular carcinoma (HCC) is a serious health concern worldwide because it is the fifth most common neoplasm and the third leading cause of cancer-related deaths globally [1]. HCC is well known to develop secondary to cirrhosis after chronic liver inflammation mainly induced by sustained infection with the hepatitis B or C viruses [2]. Recent epidemiological and experimental studies have also revealed that metabolic syndrome and obesity are critical risk factors for HCC [3]. Type 2 diabetes mellitus (T2DM) is a major complication of obesity, which has a high and increasing prevalence [4]. In addition to cardiovascular and cerebrovascular events, cancer is also a major cause of T2DM- and obesity-related deaths [5–8]. Therefore, T2DM considerably increases the risk of several kinds of malignancies, especially HCC, and the pathophysiology induced by T2DM and obesity may stimulate liver tumorigenesis [3, 9, 10].

Obesity promotes hepatic steatosis and inflammation by producing pro-inflammatory cytokines such as tumor necrosis factor (TNF)-α and interleukin (IL)-6, which are both closely related to liver carcinogenesis [10, 11]. Increased aberrant lipogenesis in the liver, which is significantly linked to obesity and metabolic syndrome, is also observed abundantly during liver carcinogenesis and HCC progression [10]. Non-alcoholic fatty liver disease (NAFLD) is considered to be closely associated with obesity and metabolic syndrome and has become one of the most common liver diseases in developed countries [12]. Furthermore, close to 20% of patients with NAFLD present with non-alcoholic steatohepatitis (NASH) with hepatocyte injury, chronic liver inflammation, and various degrees of fibrosis as well as an extensive risk of developing liver cirrhosis and HCC [13].

Recent preclinical studies have proven that therapies targeting obesity and related metabolic abnormalities such as attenuation of chronic inflammation may be an effective strategy to prevent T2DM- and NAFLD/NASH-related liver carcinogenesis [14]. For instance, treatment with pitavastatin, a drug used for hyperlipidemia, suppresses obesity- and steatosis-related liver tumorigenesis in mice by attenuating hepatic inflammation induced by excess fat deposition [15]. Metformin, an antidiabetic agent, also inhibits hepatic carcinogen diethylnitrosamine (DEN)-induced liver tumorigenesis in obese and diabetic mice [16].

Sodium-glucose cotransporter 2 (SGLT2) inhibitors have been developed for T2DM treatment and act by preventing the reabsorption of glucose filtered by the glomeruli and increasing its urinary excretion [17, 18]. This action decreases blood glucose levels and improves insulin resistance in patients with diabetes and several rodent models of T2DM [19–21]. Tofogliflozin, an SGLT2 inhibitor, has been shown to ameliorate glucose tolerance, improve dyslipidemia, and decrease liver triglyceride (TG) content in diabetic and obese animal models [22, 23]. These findings suggest that SGLT2 inhibitors might be useful agents for ameliorating metabolic abnormalities, leading to the prevention of obesity- and diabetes-related HCC development. However, detailed studies clarifying the chemopreventive effects of SGLT2 inhibitors on liver tumorigenesis have not yet been demonstrated.

Therefore, in the present study, we examined the preventive effects of tofogliflozin on DEN-induced liver tumorigenesis in C57BL/KsJ-+Lepr*^db^*/+Lepr*^db^* (*db/db*) mice, which exhibit obesity and diabetes, focusing on chronic inflammation and steatosis in the liver. We also examined the potential direct effects of tofogliflozin on human HCC cell line proliferation.

## RESULTS

### General observations

**Figure 1:**
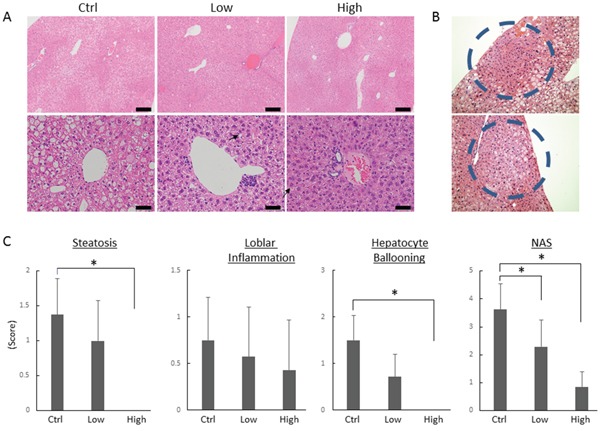
Effects of tofogliflozin on the development of pre-neoplastic lesions and histopathology in the liver of the experimental mice **(A)** Representative photomicrographs of hepatic pre-neoplastic lesions, foci of cellular alteration (FCA). **(B)** Representative photomicrographs of H&E staining at high-power field (upper panels; bars, 200 μm) and low-power field (lower panels; bars, 50 μm) of liver sections from the DEN alone-treated control mice (Ctrl), low-dose tofogliflozin-treated mice (Low), and high-dose tofogliflozin-treated mice (High) at the end of experiment. Ballooned hepatocytes are indicated by the black arrows. **(C)** Presence of NAFLD activity score (NAS; steatosis, inflammation, and ballooning) was determined using histopathological analysis. Values are expressed as mean ± SD. *P < 0.05.

The relative liver weights of the mice treated with high-dose tofogliflozin were significantly lower than those of the untreated mice at the termination of the experiment (Table 1, *P* < 0.05). Furthermore, tofogliflozin administration did not cause any clinical symptoms of toxicity. The histopathological examination also revealed that tofogliflozin did not cause toxicity in the liver, kidney, and spleen (Figure 1A and Supplementary Figure 1).

**Table 1:** Body, liver, kidneys, and white adipose tissue weights of experimental mice

Group no.	Treatment	No. of mice	Body wt (g)	Relative wt (g/100g body wt) of:		
				Liver	Kidneys	WAT^a^
1	DEN alone	8	49.6 ± 3.3^b^	4.8 ± 0.4	1.0 ± 0.1	4.6 ± 0.7
2	DEN + Tofogliflozin 1 mg/kg	7	52.0 ± 3.0	4.2 ± 0.6	1.0 ± 0.1	4.7 ± 0.5
3	DEN + Tofogliflozin 10 mg/kg	7	48.5 ± 3.5	3.5 ± 0.2^c^	1.2 ± 0.1	4.6 ± 0.4

^a^ White adipose tissue of the periorchis and retroperitoneum.

^b^ Mean ± SD.

^c^ Significantly different from group 1 by Tukey-Kramer multiple comparison test (*P* < 0.05).

### Effects of tofogliflozin on DEN-induced hepatic pre-neoplastic lesions in *db/db* mice

Foci of cellular alteration (FCA) hepatic preneoplastic lesions showing a basophilic cytoplasm and hyperchromatic nuclei (Figure 1B), were observed in the livers of DEN-administered mice at the termination of the experiment. As shown in Table 2, treatment with high-dose tofogliflozin significantly reduced the FCA incidence and multiplicity (*P* < 0.05 each) compared to those in the mice treated with DEN alone. Low-dose tofogliflozin treatment also significantly reduced the FCA multiplicity (*P* < 0.05).

**Table 2:** Effects of tofogliflozin on incidence and multiplicity of hepatic pre-neoplastic lesions in the experimental mice

Group no.	Treatment	No. of mice	Incidence	Multiplicity^b^
1	DEN alone	8	8/8 (100%)	7.6 ± 3.9^c^
2	DEN + Tofogliflozin 1 mg/kg	7	6/7 (86%)	3.9 ± 3.0^d^
3	DEN + Tofogliflozin 10 mg/kg	7	3/7 (43%)^a^	0.9 ± 0.5^d^

^a^ Significantly different from group 1 by Fisher's exact probability test (*P* < 0.05).

^b^ Number of pre-neoplastic lesions per mouse.

^c^ Mean ± SD.

^d^ Significantly different from group 1 by Tukey-Kramer multiple comparison test (*P* < 0.05).

### Effects of tofogliflozin on hepatic histopathology in the experimental mice

Hematoxylin & eosin (H&E)-stained liver sections of the experimental mice are presented in Figure 1A. Ballooning degeneration of the hepatocytes was observed in the mice treated with DEN alone, which was markedly improved by high-dose tofogliflozin. The histopathological assessment scores of the hepatic steatosis and ballooning degeneration of the hepatocytes were markedly lower in the high-dose tofogliflozin-treated mice than they were in the untreated mice (Figure 1C, *P* < 0.05). The score evaluated by the NAFLD activity score (NAS) system [24] of the high- and low-dose tofogliflozin-treated groups were also significantly lower than that of the untreated control group was (*P* < 0.05). Detailed NAS was described in Supplementary Table 1. No obvious liver fibrosis was observed in all groups in this experimental procedure.

### Effects of tofogliflozin on mRNA expression levels of pro-inflammatory markers in experimental mouse livers

**Figure 2:**
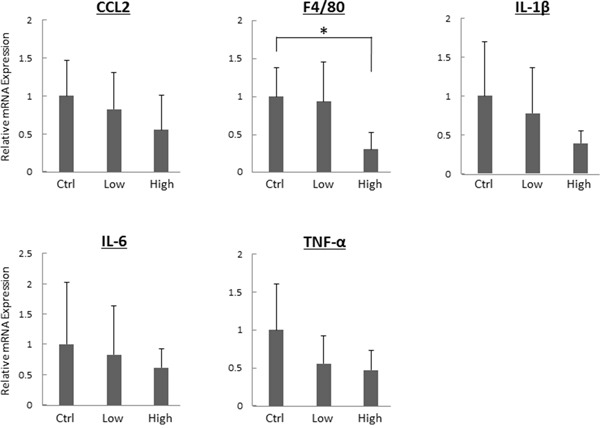
Effects of tofogliflozin on the expression levels of mRNA involved in inflammation in the liver of the experimental mice Total RNA was isolated from the livers of the experimental mice, and expression levels of mRNA associated with inflammation (*CCL2, F4/80, IL1-β, IL-6*, and *TNF-α*) were determined using quantitative real-time RT-PCR with specific primers. Values are expressed as mean ± SD. *P < 0.05.

The effects of tofogliflozin on mRNA expression levels of specific molecules associated with hepatic inflammation were examined using quantitative real-time reverse transcription-polymerase chain reaction (qRT-PCR) analysis. As shown in Figure 2, the high-dose tofogliflozin-treated mice showed a significant decrease in mRNA expression levels of macrophage marker F4/80 (*P* < 0.05). Since macrophages in the liver secrete pro-inflammatory cytokines and increase in parallel with the disease progression of mice steatohepatitis, F4/80 can be considered as a kind of marker for liver inflammation [25, 26]. Administration of high-dose tofogliflozin also decreased the mRNA expression levels of other inflammatory markers such as chemokine (C-C motif) ligand 2 (*CCL2), IL1-β, IL-6*, and *TNF-α*, but the differences were not statistically significant (*P* > 0.05).

### Effects of tofogliflozin on serum parameters and insulin resistance and sensitivity in experimental mice

As shown in Table 3, serum glucose and FFA levels were significantly decreased in tofogliflozin-treated mice (*P* < 0.05). Tofogliflozin treatment, however, did not alter serum levels of insulin, attenuate insulin resistance, or improve insulin sensitivity, which were evaluated by calculating the homeostatic model assessment of insulin resistance (HOMA-R) and the quantitative insulin sensitivity check index (QUICKI), in the present study [27, 28].

**Table 3:** Serum parameters in the experimental mice

Measurement Item	G1	G2	G3
ALT (IU/l)	104 ± 45.2	100 ± 25.1	72.6 ± 13.1
Glucose (mg/dl)	493 ± 101	364 ± 92.7	285 ± 75.4^b^
Insulin (μIU/ml)	24.5 ± 13.7	42.1 ± 27.8	54.6 ± 18.0
HOMA-R^c^	31.2 ± 21.6	37.1 ± 22.0	37.9 ± 14.3
QUICKI^d^	0.25 ± 0.01	0.25 ± 0.02	0.24 ± 0.01
Total cholesterol (mg/dl)	125.1 ± 28.3	124.6 ± 12.7	110.5 ± 20.1
FFA (μEQ/l)	1907 ± 163	1499 ± 122^b^	1494 ± 245^b^
TG (mg/dl)	114.3 ± 60.4	66.4 ± 16.6	84.5 ± 54.7

^a^ Mean ± SD.

^b^ Significantly different from group 1 by Tukey-Kramer multiple comparison test (*P* < 0.05).

^c^ HOMA-R, the homeostasis model assessment of insulin resistance.

^d^ QUICKI, quantitative insulin sensitivity check index.

### Protein expressions of SGLT2 and effects of tofogliflozin on cellular proliferation and production of cytokines in human cell lines

**Figure 3:**
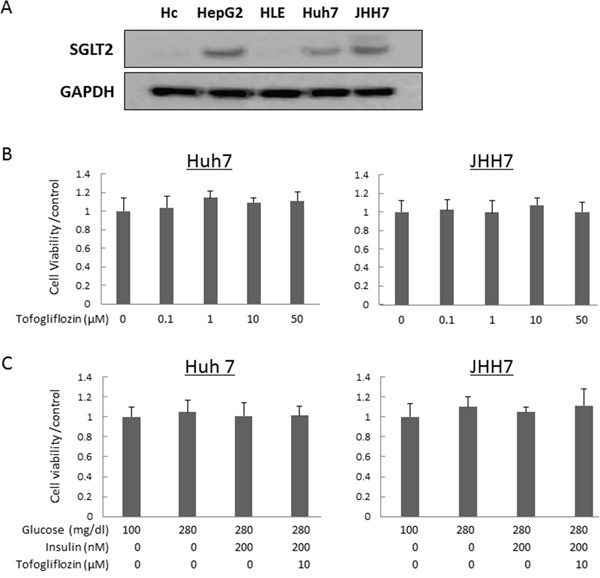
Protein expression levels of SGLT2 in human hepatocyte and hepatoma cell lines and effects of tofogliflozin on the proliferation of the HCC cells **(A)** Total protein was extracted from cultured cells and equivalent amounts of protein (10 μg/lane) were examined by western blot analysis. Primary antibodies for SGLT2 and GAPDH were used. GAPDH served as a loading control. **(B)** Cell proliferation assay of Huh7 and JHH7 cells treated with different concentrations of tofogliflozin *in vitro*. **(C)** Cell proliferation assay of Huh7 and JHH7 cells treated under normal glucose, high-glucose, high-glucose and high-insulin, and high-glucose and high-insulin plus tofogliflozin conditions. Values are expressed as mean ± SD.

To evaluate whether tofogliflozin directly inhibits the growth of HCC cells, we examined the expression levels of SGLT2 protein in four human HCC cell lines and Hc normal human hepatocytes [29, 30] using western blot analysis and performed cell proliferation assays in these cells. The SGLT2 protein was detected in HepG2, Huh7, and JHH7 cells, but the protein was not expressed in HLE and Hc cells (Figure 3A). The results of the 3-(4,5-dimethylthiazol-2-yl)- 5-(3-carboxymethoxyphenyl)- 2-(4-sulfophenyl)- 2H-tetrazolium (MTS) assay showed that both high- and low- concentrations of tofogliflozin did not inhibit the proliferation of SGLT2-expressing Huh7 and JHH7 cells (Figure 3B). Tofogliflozin treatment also did not significantly change the proliferation of these HCC cells under hyperglycemic and insulin resistance-mimicking conditions (Figure 3C). In addition, the effects of tofogliflozin on inflammatory markers were also examined in human cell lines of hepatocyte Hc and macrophage THP-1. Tofogliflozin treatment caused no significant change of the mRNA levels of cytokines, such as IL-1β and IL-6, as well in these cell lines (Supplementary Figure 2) although several significant changes were observed in the levels of these cytokines under hyperglycemic conditions.

## DISCUSSION

Obesity, a serious global health problem, and its related metabolic disorders including T2DM have been recognized as major risk factors for the development of HCC [27, 31, 32]. NAFLD is commonly associated with metabolic syndrome and can progress to NASH, which in turn leads to liver cirrhosis and HCC development [13]. Accumulating evidence indicates that NAFLD-induced cirrhosis increases the risk of HCC development in the absence of other risk factors [33]. Most NAFLD-related HCCs are believed to develop secondary to a cirrhotic liver, similar to other etiologies such as chronic hepatitis virus infection [34]. However, recent evidence also indicates that NAFLD is strongly associated with the development of non-cirrhotic HCC [35]. In such cases, the presence of metabolic syndromes, especially T2DM and obesity, may promote the development of HCC [3, 9, 10].

The results of the present study provide the first evidence that tofogliflozin, an antidiabetic SGLT2 inhibitor effectively prevents the development of the early phase of DEN-induced liver tumorigenesis in obese and diabetic *db/db* mice. SGLT2 inhibitors are a new class of oral antidiabetic drugs, which act by reducing hyperglycemia by promoting urinary glucose excretion independently of the secretion or action of insulin [36]. It has been reported that tofogliflozin, a highly selective SGLT2 inhibitor [37], reduced the hepatic TG contents in rodent models [22, 23]. Several studies have also shown that other SGLT2 inhibitors such as ipragliflozin and luseogliflozin, alleviate hepatic steatosis and steatohepatitis in rodents with obesity and T2DM [38, 39]. Consistent with these reports, the present study demonstrated that tofogliflozin significantly improved hepatic steatosis and the NAS of DEN-treated *db/db* mice. In addition, the expression levels of inflammatory markers such as F4/80 were suppressed by this agent. These findings are significant because excess lipid accumulation in the liver and subsequent inflammation accelerate hepatic tumorigenesis [11, 40, 41]. However, the improvement of steatosis and attenuation of chronic inflammation may be a key mechanism of specific agents that suppress obesity- and NAFLD/NASH-related liver carcinogenesis [28, 42, 43]. Tofogliflozin may ameliorate hepatic steatosis by decreasing serum FFA levels because the high influx of FFA into the liver strongly induces hepatic fat accumulation [11, 41].

The doses of tofogliflozin were thought to be adequate in this study because they were decided according to the data of the drug development in the animal experimentation stage, which showed sufficient antidiabetic effect without severe adverse reaction. This might account for decreasing the levels of serum glucose and FFA. In terms of insulin, the serum levels tended to be higher in tofogliflozin-treated groups although there was no statistical significance. The observation appears because tofogliflozin could preserve pancreatic β-cell function through relatively appropriate control of serum glucose level as previously reported [44], while chronic hyperglycaemia caused β-cell dysfunction and impaired insulin secretion in tofogliflozin-untreated control group. Therefore, the values of HOMA-R and QUICKI, which are calculated from the levels of serum glucose and insulin, might not be remarkably altered by the treatment with tofogliflozin.

The major limitation of our present animal experiment was that the sample size of the examination was small. In addition, carcinogen-induced cancers and gene-modified animals do not always represent human disease case precisely. Further studies are required using a large number of rodents in a proper model of the disease to investigate whether this agent exhibit similar effects as well.

Originally, SGLT2 was reported to be expressed in the renal proximal tubules [45]. However, a recent study demonstrated that certain types of cancer cells express SGLT2 and its inhibition can decrease the growth of cancer *in vivo* [46]. The present study also revealed that SGLT2 protein was expressed in several human hepatoma cell lines and, therefore, we examined whether tofogliflozin directly inhibits the growth of these cells, which possibly suppresses liver tumorigenesis. In this study, tofogliflozin did not alter the proliferation of hepatoma cell lines even under hyperglycemia- and insulin resistance-mimicking culture conditions. These findings suggest that tofogliflozin has no direct effect on the growth of HCC cells and that the suppression of tumorigenesis observed in the present study might have resulted from the amelioration of NAFLD/NASH.

In this study, the effects of SGLT2 inhibition on the expression levels of the SGLT2 were also examined *in vitro*. Wang *et al*. [47] have reported that an SGLT2 inhibitor down-regulated the mRNA levels of SGLT2 in brush border membrane. Similar to this paper, the SGLT2 mRNA levels were decreased in the human hepatoma cell HepG2 by the treatment of tofogliflozin (Supplementary Figure 3A). However, the tofogliflozin treatment had no effect on the SGLT2 levels in insulin resistance-mimicking state (Supplementary Figure 3B). These results indicate that the doses of tofogliflozin were considered to be proper in our *in vitro* study and that the inhibitor may function inadequately in insulin resistance-mimicking medium condition. Since the SGLT2 protein levels were up-regulated by SGLT2 inhibition contrary to mRNA levels [47], further studies are necessary to investigate the effects of SGLT inhibitors on the levels of the SGLTs in cancerous and non-cancerous cells.

Finally, it should be emphasized that targeting obesity and related metabolic abnormalities using pharmaceutical and nutraceutical agents may be an effective strategy for preventing liver carcinogenesis in obese individuals and antidiabetic agents, in particular, could be possible candidates for this purpose [16, 48]. Meta-analysis of observational studies showed that metformin, which can inhibit obesity- and NAFLD/NASH-related liver tumorigenesis in mice [16], is a promising agent for reducing the risk of HCC development [49]. Supplementation with branched-chain amino acids and acyclic retinoid, which exerted preventive effects on HCC development in clinical trials [32, 50], also suppressed DEN-induced liver tumorigenesis in *db/db* mice by improving hepatic steatosis and attenuating chronic inflammation [28, 42].

In summary, tofogliflozin administration appears to inhibit the progression of NAFLD and the early phase of the related liver tumorigenesis. The results of the present study further strengthen our hypothesis that targeting obesity-induced pathologies such as hepatic steatosis and chronic inflammation, may be effective for preventing liver carcinogenesis in patients who are obese and diabetic with a higher risk of HCC development [14].

## MATERIALS AND METHODS

### Animals and chemicals

Four-week-old male *db/db* mice were obtained from Japan SLC Inc., (Shizuoka, Japan) and were humanely maintained at the Gifu University Life Science Research Center in accordance with the Institutional Animal Care Guidelines. DEN was purchased from Sigma-Aldrich Chemical Corp., (St. Louis, MO, USA) while tofogliflozin was kindly provided by Kowa Co., Ltd. (Tokyo, Japan).

### Experimental procedure and histopathological examination

The animal experimental protocol was approved by the Committee of Institutional Animal Experiments of Gifu University. At 5 weeks of age, the mice were randomly divided into three experimental groups and treated as follows: DEN alone (Group 1, n = 8) and DEN plus low- and high-dose tofogliflozin (1 and 10 mg/kg in diet, Groups 2 and 3, respectively, n = 7 each). All the mice were administered tap water containing 40 ppm DEN for the first 2 weeks of the experiment. Mice in Group 1 were fed the basal diet (CRF-1, Oriental Yeast, Tokyo, Japan) throughout the experiment. At 7 weeks old, the mice in Groups 2 and 3 were fed a basal diet containing low- and high-dose tofogliflozin, respectively until the end of the experiment.

At 21 weeks old (after a 14-week tofogliflozin treatment), all the mice were euthanized to analyze the development of hepatic preneoplastic lesions FCA. Maximum sagittal sections of three sublobes (left lateral and medial and right medial lobes) were histopathologically examined. For all experimental groups, 4-μm-thick formalin-fixed and paraffin-embedded liver sections were stained with H&E for conventional histopathology. The liver histology was evaluated using NAS system [24].

### RNA extraction and qRT-PCR analysis

Total RNA was isolated from the livers of the experimental mice and cell lines as reported previously [30, 51]. qRT-PCR analysis was performed using previously reported specific primers for *CCL2*, *F4/80*, *IL-1β*, *IL-6*, *TNF-α*, and *18S* genes [51, 52]. Other primer sequences are shown in Supplementary Table 2. Each sample was analyzed using the LightCycler Nano (Roche Diagnostics, GmbH, Mannheim, Germany) with FastStart Essential DNA Green Master (Roche Diagnostics). Parallel amplification of 18S was used as the internal control.

### Clinical chemistry

Blood samples were collected from the inferior vena cava of the mice during euthanasia after an 8-hour fast and were used for chemical analyses. Enzyme immunoassay kits were used to determine serum insulin (Shibayagi, Gunma, Japan), glucose (BioVision Research Products, Mountain View, CA, USA), total cholesterol, and free fatty acid (FFA, all Wako Pure Chemical, Osaka Japan) levels according to the manufactures’ protocols. Serum alanine aminotransferase (ALT) levels were measured using a standard clinical automatic analyzer (type 7180, Hitachi, Tokyo, Japan). Insulin resistance and insulin sensitivity were determined by calculating the HOMA-R and the QUICKI, respectively [27, 28].

### Cell lines and culture conditions

The HepG2, HLE, Huh7, and JHH human hepatoma cell lines and THP-1 human macrophage cell line were obtained from the Japanese Cancer Research Resources Bank (Tokyo, Japan). The Hc human normal hepatocyte cell line was purchased from Cell Systems (Kirkland, WA, USA). All cells, except for THP-1 in RPMI-1640, were maintained in Dulbecco's modified Eagle's medium (DMEM) supplemented with 10% fetal bovine serum (FBS, both Invitrogen, Waltham, MA, USA) and 1% penicillin/streptomycin in an incubator at 37°C exposed to a humidified atmosphere of 5% CO_2_.

### Protein extraction and western blot analysis

Total protein was extracted from cultured cells, and equivalent amounts of protein (10 μg/lane) were examined using western blot as reported previously [53]. Primary antibodies against SGLT2 (sc-98975) and glyceraldehyde-3-phosphate dehydrogenase (GAPDH, #2118) are obtained from Santa Cruz Biotechnology (Santa Cruz, CA, USA) and Cell Signaling Technology (Danvers, MA, USA), respectively. GAPDH served as a loading control.

### Proliferation assays and cytokine production in cell lines

Huh7 or JHH7 cells were seeded in 96-well plates at a density of 3,000 cells. The following day, the medium was changed to a serum-free medium and the cells were treated with the indicated concentrations of tofogliflozin and glucose or insulin or both for 48 hours. The cell proliferation assays were performed using the MTS assay (Promega, Madison, WI, USA) according to the manufacturer's instructions. The cells Hc and THP-1 were treated as previously reported [30, 54] and then total RNA was isolated from the cells.

### Statistical analysis

All the data are presented as mean ± standard deviation (SD) and were analyzed using the JMP 11.0 program (Statistical Analysis Software, SAS Institute Inc., Cary, NC, USA). A one-way analysis of variance (ANOVA) was used to compare the groups. If the ANOVA indicated significant differences, the Tukey-Kramer multiple comparison test was performed to compare mean values among the groups. Fisher's exact test was used to compare the incidence of liver tumor, and all differences were considered significant at a two-sided *P*-value < 0.05.

## SUPPLEMENTARY MATERIALS FIGURES AND TABLES



## Abbreviations

ALT: alanine aminotransferase
ANOVA: analysis of variance
CCL: chemokine (C-C motif) ligand
*db/db*: C57BL/KsJ-+Lepr*^db^*/+Lepr*^db^*
DEN: diethylnitrosamine
DMEM: Dulbecco's modified Eagle's medium
FBS: fetal bovine serum
FCA: foci of cellular alteration
FFA: free fatty acid
H&E: hematoxylin and eosin
HCC: hepatocellular carcinoma
HOMA-R: homeostatic model assessment of insulin resistance
IL: interleukin
NAFLD: non-alcoholic fatty liver disease
NASH: non-alcoholic steatohepatitis
NAS: NAFLD activity score
qRT-PCR: quantitative real-time reverse transcription-polymerase chain reaction
QUICKI: quantitative insulin sensitivity check index
SGLT: sodium glucose cotransporter
TG: triglycerides
TNF: tumor necrosis factor
T2DM: type 2 diabetes mellitus.
