# Supplementary material for: Preventive effects of the sodium glucose cotransporter 2 inhibitor tofogliflozin on diethylnitrosamine-induced liver tumorigenesis in obese and diabetic mice
Source: Oncotarget. 2017 Apr 6;8(35):58353–63. doi: 10.18632/oncotarget.16874 (PMC5601657; doi:10.18632/oncotarget.16874)
Supplement: Supplementary file 1 [file oncotarget-08-58353-s001.pdf]

Preventive effects of the sodium glucose cotransporter 2 inhibitor tofogliflozin on diethylnitrosamine-induced liver tumorigenesis in obese and diabetic mice

SUPPLEMENTARY FIGURES AND TABLES

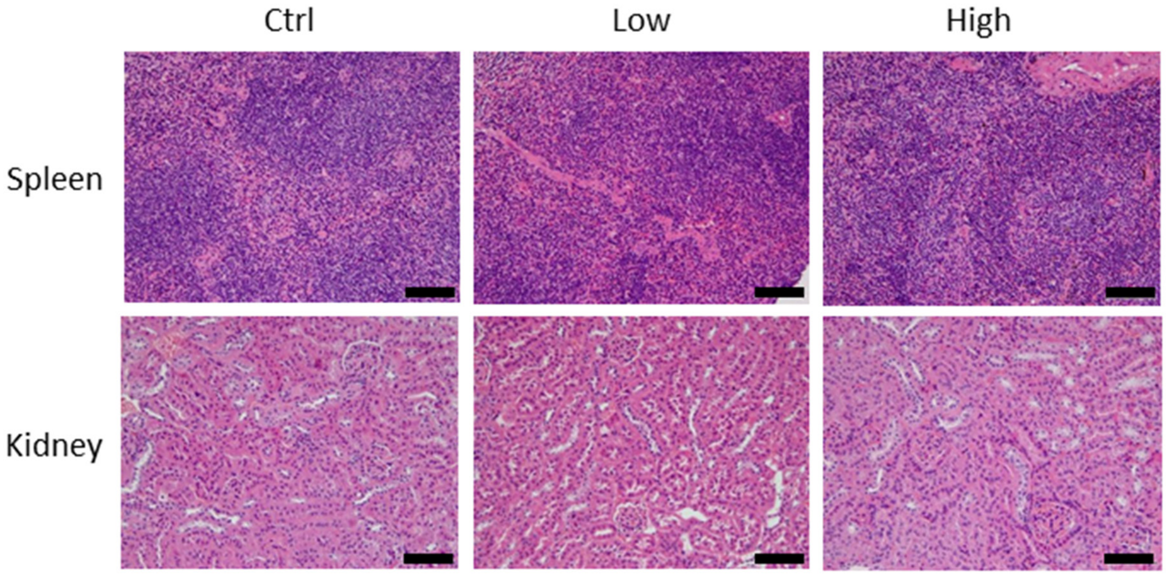

Supplementary Figure 1: Histopathology in the spleen and kidney of the experimental mice. Representative photomicrographs of H&E staining of spleen and kidney sections from the DEN alone-treated control mice (Ctrl), low-dose tofogliflozin-treated mice (Low), and high-dose tofogliflozin-treated mice (High) at the end of experiment. Bars, 100 µm.

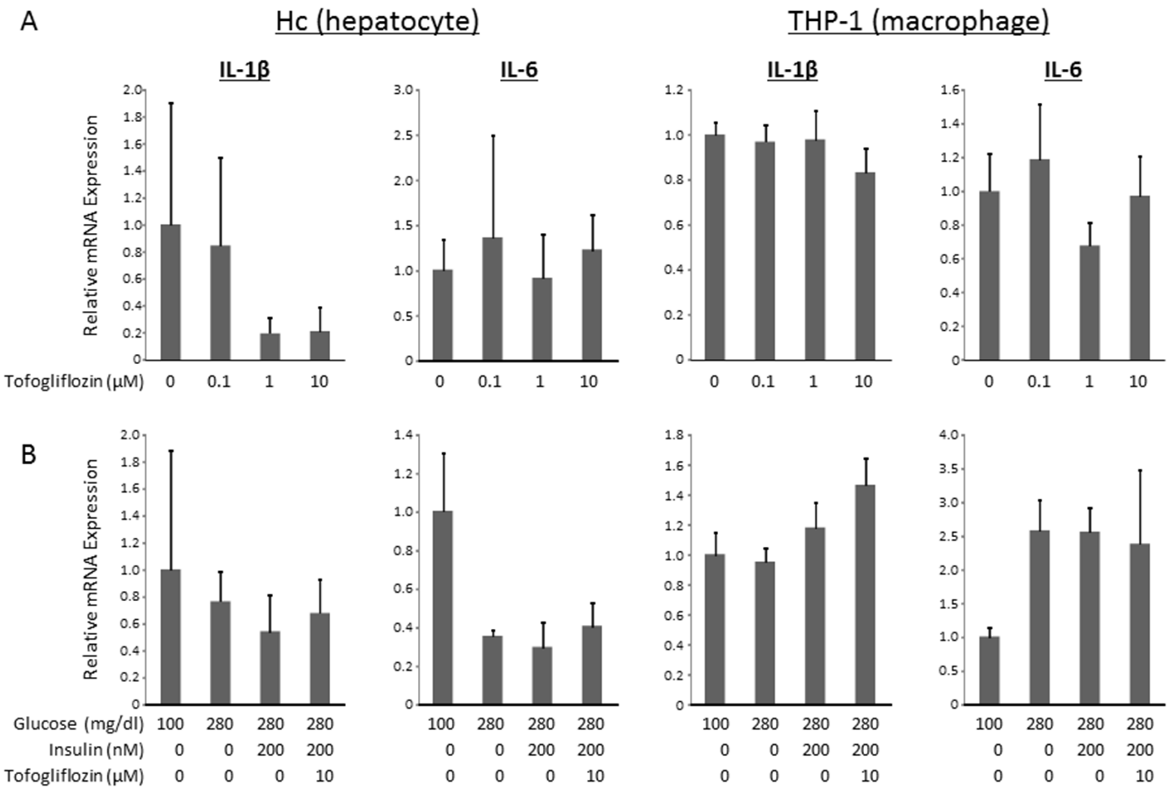

Supplementary Figure 2: Effects of tofogliflozin on the expression levels of mRNA related to inflammation in human hepatocyte and macrophage cell lines. **(A)** Human cell lines, hepatocyte Hc and macrophage THP-1, were treated with different concentrations of tofogliflozin *in vitro*. **(B)** Cells were also treated under normal glucose, high-glucose, high-glucose and high-insulin, and high-glucose and high-insulin plus tofogliflozin conditions. Total RNA was isolated from cells and expression levels of mRNA associated with inflammation, including IL1-β and IL-6, were determined using quantitative real-time RT-PCR with specific primers. Values are expressed as mean ± SD.

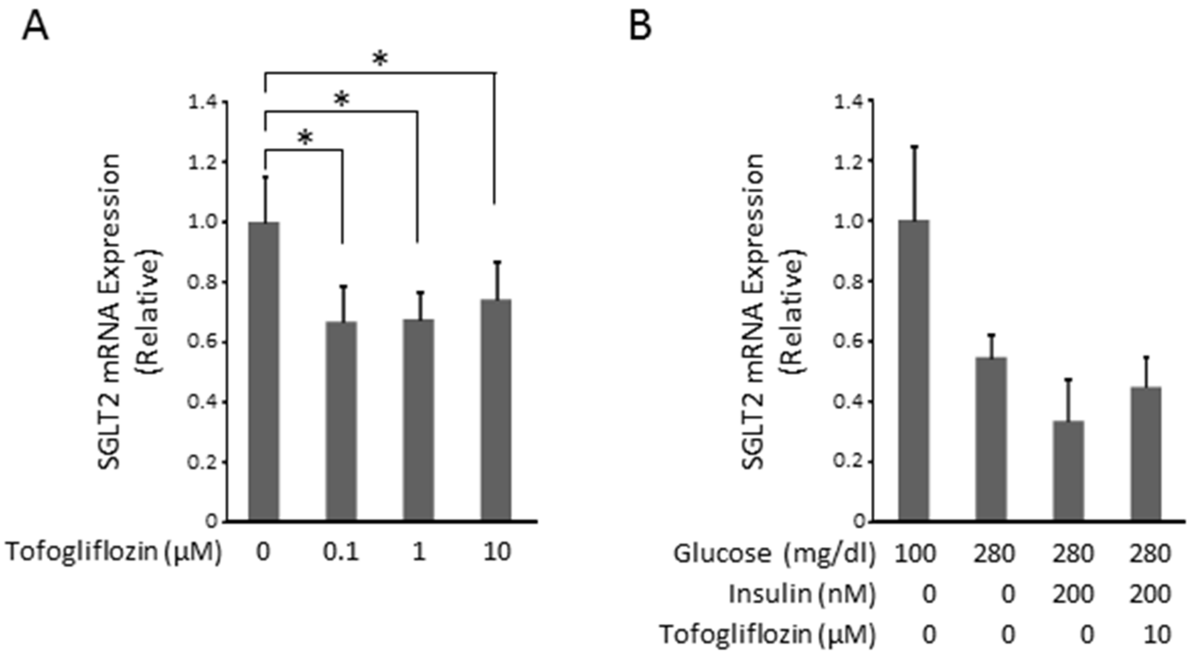

Supplementary Figure 3: Effects of tofogliflozin on the expression levels of SGLT2 mRNA in the human hepatoma cell line. **(A)** The human hepatoma cells HepG2 were treated with different concentrations of tofogliflozin *in vitro*. **(B)** Cells were also treated under normal glucose, high-glucose, high-glucose and high-insulin, and high-glucose and high-insulin plus tofogliflozin conditions. Total RNA was isolated from cells and expression levels of SGLT2 mRNA were determined using quantitative real-time RT-PCR with the specific primer. Values are expressed as mean ± SD. $^{*}P < 0.05$.

Supplementary Table 1: NAFLD activity scores in each mouse

	Mouse no.	Score		
		Steatosis	Lobular inflammation	Hepatocyte ballooning
Group 1 (Ctrl)	1	2	0	2
	2	1	0	2
	3	2	1	2
	4	1	1	1
	5	2	1	2
	6	1	1	1
	7	1	1	1
	8	1	1	1
Group 2 (Low)	1	1	1	0
	2	1	1	1
	3	1	0	1
	4	1	0	1
	5	2	1	1
	6	1	0	1
	7	0	1	0
Group 3 (High)	1	0	1	0
	2	0	0	0
	3	0	1	0
	4	0	0	0
	5	0	1	0
	6	0	0	0
	7	0	0	0

Supplementary Table 2: Primer sequences for *in vitro* experiment

Gene (human)	Primer sequences (5'-3')	
	Forward	Reverse
IL-1β	TACCTGTCCTGCGTGTTGAA	TCTTTGGGTAATTTTTGGGATCT
IL-6	CAGGAGCCCAGCTATGAACT	GAAGGCAGCAGGCAACAC
SGLT2	GTTGCTGGATTCGAGTGGA	AGGTACACGGGTGCAAACA
18S	GCAATTATTCCCCATGAACG	GGGACTTAATCAACGCAAGC
